# MIKE: an ultrafast, assembly-, and alignment-free approach for phylogenetic tree construction

**DOI:** 10.1093/bioinformatics/btae154

**Published:** 2024-03-28

**Authors:** Fang Wang, Yibin Wang, Xiaofei Zeng, Shengcheng Zhang, Jiaxin Yu, Dongxi Li, Xingtan Zhang

**Affiliations:** College of Computer Science and Technology, Taiyuan University of Technology, Taiyuan, Shanxi 030024, China; National Key Laboratory for Tropical Crop Breeding, Shenzhen Branch, Guangdong Laboratory for Lingnan Modern Agriculture, Genome Analysis Laboratory of the Ministry of Agriculture, Agricultural Genomics Institute at Shenzhen, Chinese Academy of Agricultural Sciences, Shenzhen, Guangdong 518120, China; National Key Laboratory for Tropical Crop Breeding, Shenzhen Branch, Guangdong Laboratory for Lingnan Modern Agriculture, Genome Analysis Laboratory of the Ministry of Agriculture, Agricultural Genomics Institute at Shenzhen, Chinese Academy of Agricultural Sciences, Shenzhen, Guangdong 518120, China; Department of Human Cell Biology and Genetics, Joint Laboratory of Guangdong-Hong Kong Universities for Vascular Homeostasis and Diseases, School of Medicine, Southern University of Science and Technology, Shenzhen, Guangdong 508055, China; National Key Laboratory for Tropical Crop Breeding, Shenzhen Branch, Guangdong Laboratory for Lingnan Modern Agriculture, Genome Analysis Laboratory of the Ministry of Agriculture, Agricultural Genomics Institute at Shenzhen, Chinese Academy of Agricultural Sciences, Shenzhen, Guangdong 518120, China; National Key Laboratory for Tropical Crop Breeding, Shenzhen Branch, Guangdong Laboratory for Lingnan Modern Agriculture, Genome Analysis Laboratory of the Ministry of Agriculture, Agricultural Genomics Institute at Shenzhen, Chinese Academy of Agricultural Sciences, Shenzhen, Guangdong 518120, China; College of Computer Science and Technology, Taiyuan University of Technology, Taiyuan, Shanxi 030024, China; National Key Laboratory for Tropical Crop Breeding, Shenzhen Branch, Guangdong Laboratory for Lingnan Modern Agriculture, Genome Analysis Laboratory of the Ministry of Agriculture, Agricultural Genomics Institute at Shenzhen, Chinese Academy of Agricultural Sciences, Shenzhen, Guangdong 518120, China

## Abstract

**Motivation:**

Constructing a phylogenetic tree requires calculating the evolutionary distance between samples or species via large-scale resequencing data, a process that is both time-consuming and computationally demanding. Striking the right balance between accuracy and efficiency is a significant challenge.

**Results:**

To address this, we introduce a new algorithm, MIKE (MinHash-based *k*-mer algorithm). This algorithm is designed for the swift calculation of the Jaccard coefficient directly from raw sequencing reads and enables the construction of phylogenetic trees based on the resultant Jaccard coefficient. Simulation results highlight the superior speed of MIKE compared to existing state-of-the-art methods. We used MIKE to reconstruct a phylogenetic tree, incorporating 238 yeast, 303 *Zea*, 141 *Ficus*, 67 *Oryza*, and 43 *Saccharum spontaneum* samples. MIKE demonstrated accurate performance across varying evolutionary scales, reproductive modes, and ploidy levels, proving itself as a powerful tool for phylogenetic tree construction.

**Availability and implementation:**

MIKE is publicly available on Github at https://github.com/Argonum-Clever2/mike.git.

## 1 Introduction

The swift evolution of next-generation sequencing technology (Van Dijk *et al.* 2014, Goodwin *et al.* 2016) has driven a steady decrease in sequencing costs, thereby facilitating the production of large-scale high-throughput sequencing data (Metzker 2010, De Coster *et al.* 2021). A prime example of this is the comprehensive rice pan-genome project, which sequenced over 3000 rice samples (Zhao *et al.* 2018). Phylogenetic trees, which delineate the evolutionary relationships among biological entities, are vital for studying life’s evolution (Bos and Posada 2005, Ronquist *et al.* 2009). However, the data analysis needed for inferring these trees is still a labor-intensive and computationally challenging task (Kapli *et al.* 2020). Given the escalating volume of data, the demand for efficient and precise methods for constructing phylogenetic trees is urgent. While some related methods, such as CallSNPs (Morin *et al.* 2004, Batley and Edwards 2007), is prevalent for constructing these trees, it requires substantial computational resources and depends on a reference genome (Alexandrov *et al.* 2015), which many species lack.

Ondov *et al.* acknowledged the potential of the minhash technique (Buhler 2001), primarily for its minimal memory usage and exceptional speed. They developed Mash (Ondov *et al.* 2016), a tool capable of calculating the Jaccard coefficient (Niwattanakul *et al.* 2013) and the Mash evolutionary distance. The Jaccard coefficient is used to calculate the similarity between samples, and the Mash evolutionary distance can be considered an estimate of the mutation rate. It can be employed, through hierarchical clustering, to infer the evolutionary relationships between species and construct a phylogenetic tree. However, the Mash evolutionary distance is closely related to the size of the sketch. This relationship can potentially impact the accuracy of phylogenetic tree construction, leading to the possibility of incorrect branches.

BinDash (Zhao 2019) is another method used for calculating the Jaccard coefficient between genomes. It combines approaches like b-bit minhash and one-permutation minhash (Shrivastava 2017). Notably, BinDash significantly reduces memory consumption. However, it does not explicitly mention its application in constructing phylogenetic trees. Kssd (Yi *et al.* 2021) is also based on the minhash algorithm used to calculate the Jaccard coefficient. It selects suitable k-mers through feature subspaces. While Kssd has shown improvement in speed compared to Mash and BinDash, it requires a larger operating memory space and is not intended for constructing phylogenetic trees. Skmer (Sarmashghi *et al.* 2019) and AAF (Fan *et al.* 2015) are also methods that can be used for constructing phylogenetic trees without assembly, but their speed needs improvement. Currently, there is a gap in the availability of a fast and efficient tool that can balance speed and accuracy for the construction of phylogenetic trees.

In response to these challenges, we introduce MIKE (MinHash-based k-mer algorithm), an approach specifically designed for the rapid computation of the Jaccard coefficient and the Mash evolutionary distances. It constructs phylogenetic trees using the computed distance matrix through the BIONJ (the bio-Neighbor Joining) (Gascuel 1997) or NJ (the Neighbor Joining) (Saitou and Nei 1987) approach. MIKE bypasses genome assembly and alignment requirements and exhibits exceptional data processing capabilities, efficiently handling large datasets in a short timeframe. Compared to the traditional CallSNPs method, the runtime is significantly reduced. In comparison to other methods based on raw sequence data, not only is the runtime significantly decreased, but the accuracy is also notably improved.

## 2 Materials and methods

### 2.1 Simulation dataset

In generating simulated data for constructing the phylogenetic tree, we applied the Jukes–Cantor model (Erickson 2010), which assumes equal probabilities for all nucleotide substitutions. In addition, these substitutions occur in specified regions, simulating genomic areas with higher mutation rates, namely mutation regions, while the remaining regions remain unchanged, representing conserved regions. The genome undergoes mutations at a certain mutation rate, leading to the emergence of new genomes considered as offspring. In each generation, they give rise to new genomes through mutations. Subsequently, all descendant genomes from the four types of datasets were further simulated as raw sequencing data using Art_illumina (Huang *et al.* 2012).

**Figure 1.**
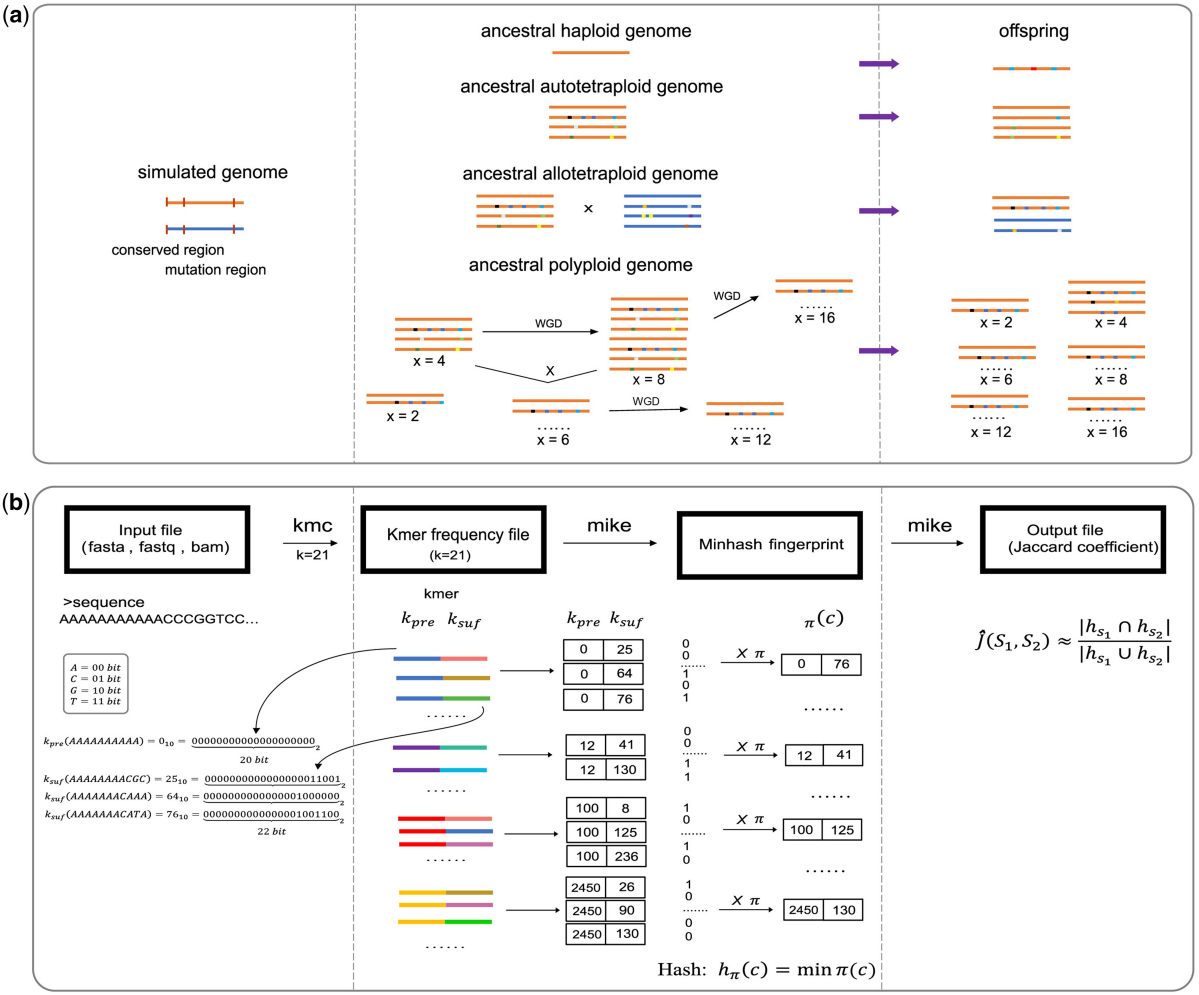
The process of simulating data and overview of MIKE algorithm. (a) The process of simulating data. Four sets of monoploid datasets were simulated, including haploid, autotetraploid, allotetraploid, and polyploid. Beginning with the same ancestral chromosomes, some regions are designated as conservative (no mutations) and others as non-conservative (allowing mutations). Polyploids can generate new genomes through processes such as whole-genome duplication (WGD) and hybridization. The polyploid datasets were simulated with six ploidy levels: diploid, tetraploid, hexaploid, octoploid, dodecaploid, and hexadecaploid. Each dataset is designed to undergo mutations in each generation, resulting in the generation of *n* different offspring in the process. (b) The overview of MIKE algorithm. First, the sequencing reads are divided into *k*-mers, with *k* set to 21 by default. A mapping is defined to represent each character using 2 bits, where A, C, G, and T correspond to 00, 01, 10, and 11, respectively. Each *k*-mer is split into two parts, defined as a prefix and a suffix, $k_{\mathrm{pre}}$ and $k_{\mathrm{suf}}$. Subsequently, *k*-mers with the same $k_{\mathrm{pre}}$ are grouped together. Within each group, a random shuffled permutation π with a numerical range of $\left[1,\;\max(k_{\mathrm{suf}})\right]$ is applied. All $k_{\mathrm{suf}}$ values for each group are marked with either 0 or 1 to create one-hot vectors, where a value is marked as 1 if the $k_{\mathrm{suf}}$ occurs and 0 if it does not. These vectors are then multiplied by the permutation π and the smallest non-zero value $h_{\pi }(c)$ is selected as the representative feature value for that group, known as the minhash fingerprint. This minhash fingerprint can effectively represent the original sequencing data

As shown in Fig. 1a, for haploid data, it follows the process of generating offspring through repeated mutations. For autotetraploid data, two chromosomes are randomly chosen, mutations are introduced in mutation regions, and then, with these chromosomes as parental, the process of autotetraploid variation is repeated, giving rise to new autotetraploid genomes. For allotetraploid data, two chromosomes were independently chosen from the ancestral genomes of two tetraploids, serving as the paternal parent and maternal parent. In each generation, mutations occurred, and two offspring genomes were produced through hybridization. This process was iterated until a total of 2*n* offspring genomes were obtained.

For polyploid data, we simulated an ancestral genome with a ploidy level of *x* = 2, and genome with a ploidy level of *x* = 4. The genome with a ploidy level of *x* = 4 underwent whole-genome duplication (WGD), resulting in the emergence of genomes with ploidy levels of *x* = 8. The *x* = 8 also experienced WGD to yield a ploidy level of *x* = 16. The *x* = 4 and *x* = 8 could hybridize, leading to a genome of *x* = 6, and the *x* = 6 could undergo WGD events as well. The process of mutation, hybridization, and WGD was repeated over generations. In each generation, datasets with offspring genomes of ploidy levels *x* = 2, 4, 6, 8, 12, and 16 were generated.

### 2.2 Overview of MIKE algorithm

Each read can be divided into a collection of *k*-length substrings known as *k*-mers, achieved by sliding a window of length *k* along the sequence. All *k*-mers are sorted in lexicographic order, using the built-in KMC (Kokot *et al.* 2017) tool. For the set Σ={A, C, G, T}, a mapping is defined to represent each character using 2 bits, where A, C, G, T correspond to $00_{2}$, $01_{2}$, $10_{2}$, $11_{2}$, respectively. Each *k*-mer is split into two parts, defined as a prefix $k_{\mathrm{pre}}$ and a suffix $k_{\mathrm{suf}}$. The length of $k_{\mathrm{pre}}$ is $2\times \left\lfloor k/2\right\rfloor \;\mathrm{bits}$, the length of $k_{\mathrm{suf}}\;$is $2\times \left\lceil k/2\right\rceil$ bits, as shown in Fig. 1b. For any arbitrary shuffled permutation π, it represents a shuffled array ranging from 1 to $\max(k_{\mathrm{suf}})$. All identical prefixes $k_{\mathrm{pre}}\;$are grouped together, and for each group, define a one-hot vector that sequentially labels the occurrence of suffixes within that group. If an occurrence of $k_{\mathrm{suf}}\;$exists, the right site of the one-hot vector is marked as 1; if it does not exist, the right site of the one-hot vector is marked as 0. The combination of one-hot vectors from all groups forms an existence matrix, consist of two elements, 0 and 1. The size of this matrix is the number of all groups multiplied by the maximum value of suffixes. Subsequently, the existence matrix is then multiplied by the random permutation π, and the first non-zero value is selected as the minhash fingerprint. Ultimately, this process reduces the dimensionality of data from a maximum of $4^{21}$–$4^{10}$.

This method effectively compresses data while preserving essential information for sequence data. MIKE relies on sorted *k*-mers, and during data processing, it only needs to linearly process the data from the beginning to the end, with a time complexity of *O*(*n*). MIKE employs a grouping method that is tantamount to dimensionality reduction for each group, with the objective of preserving the characteristic hash values of each group as effectively as possible. The calculations involve an existing matrix and make use of a random permutation function shared across all groups. In contrast, Mash directly hashes the entire dataset and selects hashed values based on the sketch size. The calculation of the approximate Jaccard coefficient is influenced by the sketch size. Even if the specified sketch size is the same as the number of groups in MIKE, there still exists a preference for locality-sensitive hashing in Mash, which introduces certain biases in the results.

### 2.3 Evolutionary distance

We use the Jaccard coefficient to calculate the evolutionary distance between two species. Given two genomes, denoted as $S_{1}$ and $S_{2}$, originating from a nucleotide alphabet set $\Sigma =\left\{A,\;C,\;G,\;T\right\}$. The lengths of $S_{1}$ and $S_{2}$ are both L. All sequences within both datasets are partitioned into subsequences of size k. The Jaccard coefficient is the ratio of the intersection to the union of two sets, used to measure the similarity and dissimilarity between finite sample sets, defined as

$$J\left(S_{1},\;S_{2}\right)=\frac{\left|S_{1}\cap S_{2}\right|}{{|S}_{1}\cup S_{2}|}$$

The number of *k*-mer shared between two sets is denoted as W.

$$J\left(S_{1},\;S_{2}\right)=\frac{\left|S_{1}\cap S_{2}\right|}{{|S}_{1}\cup S_{2}|}=\frac{W}{2L-W}$$

which can be obtained as $\frac{W}{L}=\frac{2J}{J+1}$.

For a particular nucleotide, the probability of mutating to another nucleotide is denoted as d. Assuming that only single base substitutions occur in the genome, which follows the Poisson distribution, then for a *k*-mer of length k, the probability of it remaining unchanged is given by

$$p={\left(1-d\right)}^{k}$$

Since the occurrence of each *k*-mer is a random independent event, following the Bernoulli trial, and the relationship can be expressed as:

$$\frac{W}{L}={\left(1-d\right)}^{k}\to d=1-{\left(\frac{2J}{J+1}\right)}^{\frac{1}{k}}$$

The Jaccard coefficient approximate value is obtained through MIKE to calculate the evolutionary distance.

$$d=1-{\left(\frac{2\hat{j}\left(S_{1},\;S_{2}\right)}{\hat{j}\left(S_{1},\;S_{2}\right)+1}\right)}^{\frac{1}{k}}$$

## 3 Result

### 3.1 Accuracy of resemblance estimation

To assess the accuracy of MIKE in calculating the Jaccard coefficient, we considered the impact of sequencing coverage and the values of *k* on its performance. The genome of *Drosophila melanogaster* (Berlin *et al.* 2015) is utilized as the source for all simulated data generated through art_illumina and Seqkit (Shen *et al.* 2016). The Jaccard coefficient estimated is calculated using methods like Mash, BinDash, and Kssd, and compared with MIKE. The ground truth of the Jaccard coefficient was determined by the ratio of the intersection to the union of all *k*-mers. The root mean square error (RMSE) is a commonly used statistic for measuring the difference between the approximate values and the ground truth. Smaller RMSE values indicate better predictive model performance.

#### 3.1.1 Evaluation of accuracy with simulated data at same sequence coverage

For simulated sequencing data ranging from 10× to 90× coverage, as shown in Supplementary Fig. S1, the RMSE deviation between MIKE and the ground truth is consistently below 0.01, with some results even falling below 0.001. The RMSE exhibits only marginal variance between MIKE and the ground truth. Additionally, the Jaccard coefficient calculated by MIKE is consistently above 0.9 in Supplementary Fig. S2. In contrast, the other three methods, the results of Mash, BindDash, and Kssd are inferior to those of MIKE.

#### 3.1.2 Evaluation of accuracy with simulated data at various sequence coverage

As shown in Supplementary Fig. S3, spanning from 0.5× to 100× coverage, the Jaccard coefficient exhibited its optimal performance within the range of 10× to 20×, with similarity consistently at approximately 0.97. This result indicates that higher sequence coverage is not necessarily required for accurate Jaccard coefficient calculation. Among the four methods, when the sequence coverage is below 4×, the performance of the other three methods surpasses that of MIKE. Nevertheless, as we move into the sequence coverage ranging from 4× to 100×, MIKE consistently demonstrates superior performance.

At low sequence coverages, the coverage range of sequencing data is relatively limited, potentially resulting in the omission of numerous *k*-mers and an increased presence of missing information and noise. Conversely, at high sequence coverages, the data from sequencing errors in large-scale samples may increase, potentially leading to slight distortions in the calculation of certain *k*-mers. Consequently, in both low and high sequence coverage, the results of the Jaccard coefficient are compromised. Moreover, based on the above results, it can be inferred that the impact on the Jaccard coefficient is smaller at the same sequence coverage than at different sequence coverage.

#### 3.1.3 Evaluation of accuracy with simulated data at various *k* values

We selected 10 sets of data with the sequence coverage of 40×, and set *k* values to 17, 19, 21, 25, and 27, respectively. As shown in Supplementary Fig. S4, the ground truth exhibited a continuous upward trend with increasing *k* values. At *k* = 21, a peak was reached, followed by a decline. This trend is speculated to be a result of the heightened influence of variations and sequencing errors due to the increased *k* values, amplifying the impact of base changes on calculating sequence similarity. MIKE’s results consistently increase with the increase in *k* values, leading to a growing deviation from the standard values of the true dataset. Through the comparison of different *k* values, *k* = 21 was ultimately chosen as the foundational setting for subsequent research.

#### 3.1.4 Computational efficiency

**Figure 2.**
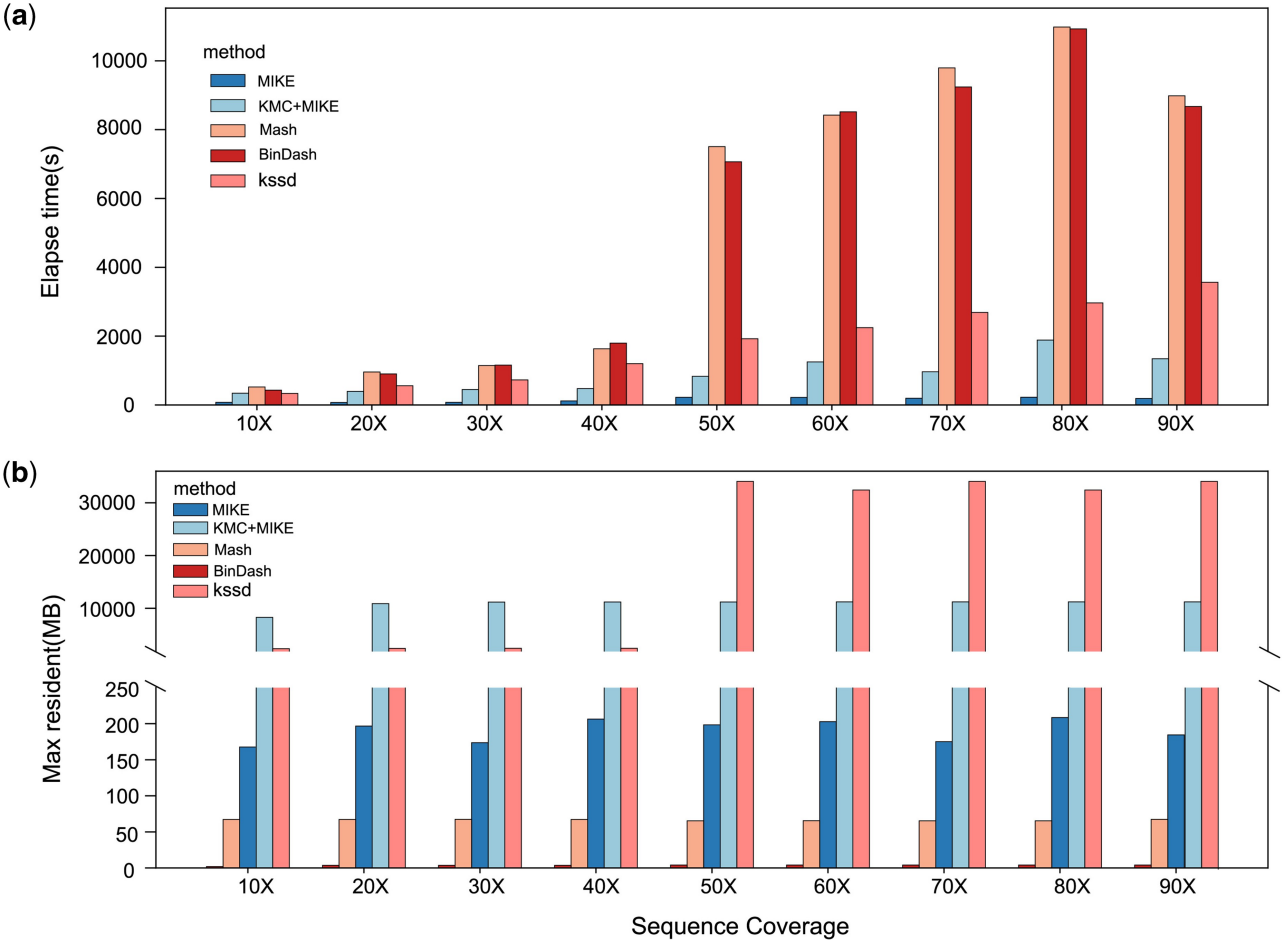
Computational efficiencies. (a) The process of simulating data. (b) Four sets of monoploid datasets were simulated, including haploid, autotetraploid, allotetraploid, and polyploid

We evaluated the elapsed time and peak resident memory usage of four tools based on the minhash algorithm. Mash exhibited the longest execution time in Fig. 2. The elapsed time of BinDash was nearly identical to those of Mash, but it had the lowest maximum memory usage, approximately 4 MB. When comparing the four methods, Kssd slightly lags behind MIKE in terms of speed. However, beyond a coverage of 50×, Kssd’s memory consumption spikes, exceeding 30 000 MB. This significant increase in memory usage is primarily attributed to the need to create an enlarged subspace to record all acquired *k*-mers.

Focusing on MIKE’s processing of the *k*-mer frequency file, it boasts the shortest processing time among all evaluated methods. This holds true even when considering the additional step of using KMC to generate the *k*-mer frequency file from the resequencing data (MIKE + KMC). In terms of memory usage, MIKE’s peak memory usage is around 200 MB, slightly higher than Mash and BinDash’s peak memory usage but still far lower than Kssd. Even when considering KMC, the memory consumption of MIKE remains significantly lower than Kssd, especially at sequence coverage exceeding 50×.

### 3.2 Selection of parameters for phylogenetic tree construction

To assess the impact of different parameters on the phylogenetic tree topology, we selected 19 species from the *Anopheles* genus to construct phylogenetic trees. According to the evolutionary distance formula d, different *k* values affect d, and using different distance methods also influences the shape of the tree. We chose *k* values of 17, 19, 21, 25, and 27, respectively, constructing phylogenetic trees for 19 species of the *Anopheles* genus, with the intermediate value *k* = 21 serving as the baseline for comparison. Four common distance methods for tree construction, namely UPGMA (Unweighted Pair Group Method with Arithmetic Mean) (Paradis and Schliep 2019), NJ (Neighbor-Joining Tree Estimation), BIONJ (Tree estimation Based on an Improved Version of the NJ Algorithm), and the Minimum Evolution method, were employed. We constructed phylogenetic trees for 22 *Ficus* species and 67 *Oryza* species using each of the four distance methods, with the results from CallSNPs used as the reference for comparison. The generalized Robinson–Foulds distance (RF distance) (Smith 2020) is the metric for assessing topological consistency in the phylogenetic trees. A larger RF distance indicates a poorer match between the results.

#### 3.2.1 Selection of *k* value

As shown in Supplementary Fig. S5, with the increase in *k* values, the topology of the phylogenetic tree tends to stabilize. At *k* = 17, its RF distance from *k* = 21 is 0.05. Although the RF distance is 0 when *k* = 19, there is a miscalculation on the branch of *Anopheles nili*, resulting in minor errors. When *k* = 25 and *k* = 27, the phylogenetic tree topology is completely consistent with that of *k* = 21, aligning with the previously discussed conclusion regarding the Jaccard coefficient.

#### 3.2.2 Different distance methods for constructing phylogenetic trees

In the *Ficus* genus, the RF distances of phylogenetic trees constructed using NJ, BIONJ, and the Minimum Evolution method compared to those constructed using CallSNPs are all 0.21, while the tree constructed using UPGMA shows even smaller differences with an RF distance of 0.15 in Supplementary Fig. S6. In the *Oryza* genus, the RF distances for UPGMA and the Minimum Evolution are 0.37, slightly worse than the results of NJ and BIONJ in Supplementary Figs S7 and S8. It suggests that specific species may require the selection of different methods based on their characteristics for tree construction. To ensure the consistency of results, we used NJ or BIONJ for constructing phylogenetic trees in both simulated and real datasets.

### 3.3 Construction of phylogenetic tree

#### 3.3.1 Application on phylogenetic analysis with simulated data

To evaluate the potential of using MIKE for phylogenetic tree construction, four sets of simulated datasets are generated based on an initial simulated genome with introduced variations. Reproductive mode and ploidy level were considered critical factors, given their potential impact on phylogenetic tree construction. The four sets of datasets simulate approximately 1000 generations of natural evolution. (as detailed in the Section 2). MIKE, Mash, BinDash, and Kssd are employed to calculate the Jaccard coefficient and obtain distance matrices. Additionally, CallSNPs is utilized with VCF2Dis (Dong *et al.* 2021) to generate distance matrices for constructing the phylogenetic tree. The accuracy of various tools was then compared.

As shown in Supplementary Figs S9–S13, in the four simulated datasets, the results of MIKE and CallSNPs are generally similar. For haploid simulated data, autotetraploid, and allotetraploid, their RF distances are all below 0.21, with slightly poorer performance in the polyploid dataset. The other three methods do not perform as well as MIKE and are not suitable for constructing phylogenetic trees. In contrast to CallSNPs, MIKE has demonstrated greater efficiency and speed in constructing phylogenetic trees. Across all the assembly- and alignment-free methods employed, MIKE demonstrated superior stability, consistency, and accuracy. This performance was consistent across various simulated datasets, confirming MIKE as a reliable choice for phylogenetic tree construction.

#### 3.3.2 Application on phylogenetic tree construction with real data

##### 3.3.2.1 Phylogenetic trees based on different evolutionary distances

To assess the robustness of MIKE in constructing phylogenetic trees based on evolutionary distances, we conducted an extensive and in-depth empirical evaluation. Twenty-six distinct plant species were selected, representing a diverse range of evolutionary distances spanning across lineages, covering a broad spectrum from *Chlorophyta* to *Magnoliophyta*, including Poaceae, Brassicaceae, and Selaginellaceae. In the evolutionary process of plants, such as ferns and clubmosses are generally considered to be more advanced than mosses (Dylus *et al.* 2023). As shown in Supplementary Fig. S14, with *Chlorella variabilis* as the root, there is a certain deviation in the branching relationship between *Selaginella moellendorffii* and *Physcomitrella patens*, but the overall classification is mostly accurate. Species of the Poaceae, Fabaceae, Brassicaceae, Solanaceae, and Malvaceae families all form separate branches without classification errors.

Subsequently, data from 238 yeast strains were also used to construct a phylogenetic tree. As shown in Supplementary Fig. S15, the dataset encompasses seven distinct families within the subphylum *Saccharomycotina*. A comparative analysis with Shen *et al.* (2018) revealed significant discrepancies, particularly concerning the performance of *Pichiaceae* and *Saccharomycetaceae*. In the phylogenetic tree constructed by MIKE, what was anticipated as a monophyletic branch displayed multiple branching. MIKE is not effective for robust classification in yeast. However, if only considering a specific family, such as the *Metschnikowia* family in Supplementary Fig. S16, MIKE’s classification is able to reproduce the correct classification.

**Figure 3.**
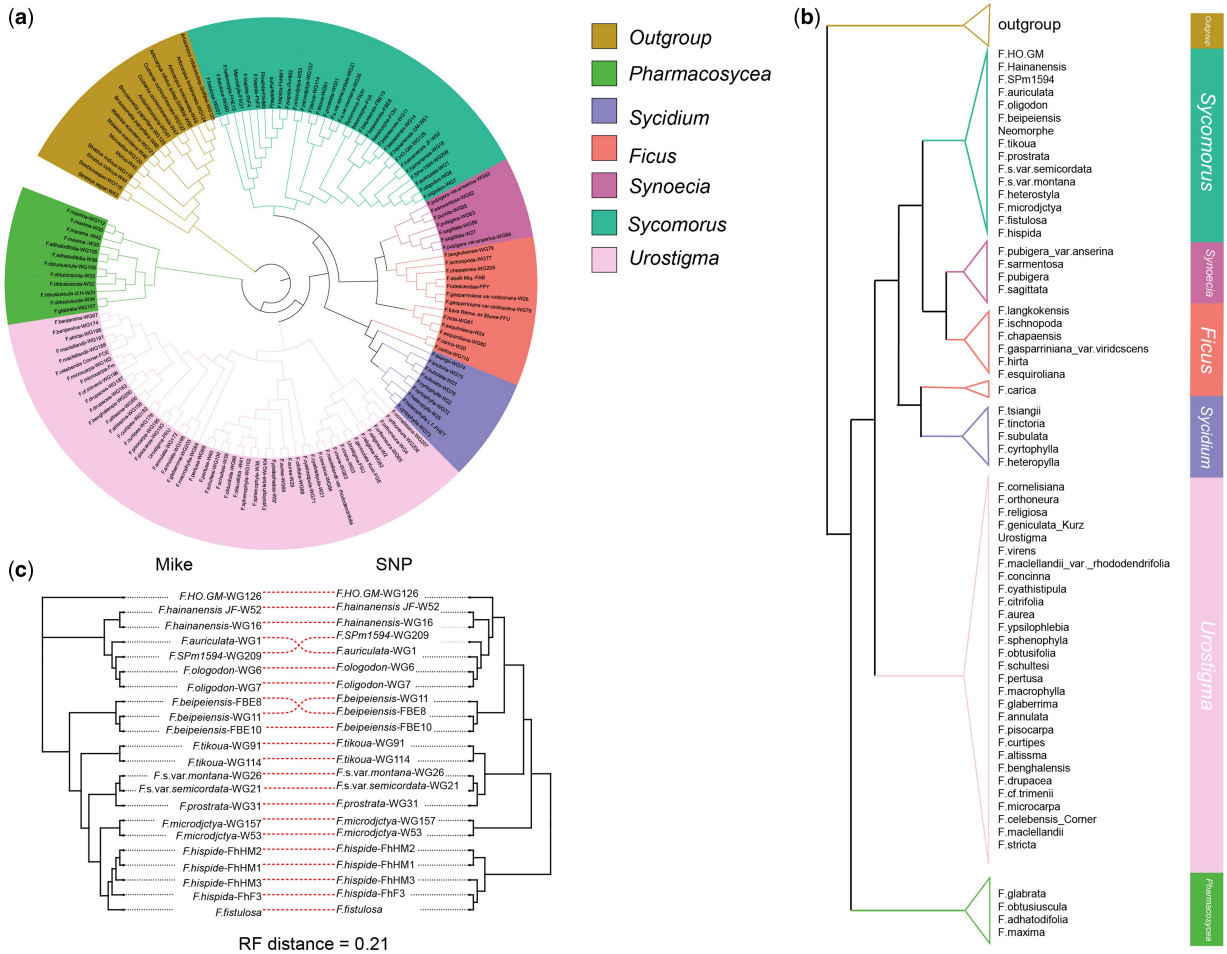
The phylogenetic tree of Ficus. (a, b) The phylogenetic tree constructed using MIKE for 141 samples of the genus *Ficus*. (c) The phylogenetic tree for 22 species selected from the subgenus *Sycomorus* of the genus *Ficus*, constructed using both MIKE and CallSNPs through BIONJ methods

Our research has shifted toward intrageneric studies, incorporating species with larger genomes. In Fig. 3a and b, we constructed a dataset for *Ficus*, comprising 141 samples, including six subgroups and one outgroup. Similar to previous results (Zhang *et al.* 2020), the subgenus *Ficus* is distinctly divided into two groups. The divergence times of these six subgenera follow the commonly accepted *Ficus* phylogeny sequence, with *Pharmacosycea*, *Urostigma*, *Sycidium*, *Ficus*, *Synoecia*, and *Sycomorus*. To further compare the topologies within the subgenus, we randomly selected 22 species from the *Sycomorus* subgenus and constructed phylogenetic trees using both MIKE and CallSNPs. As shown in Fig. 3c, the RF distance between them was 0.21.

**Figure 4.**
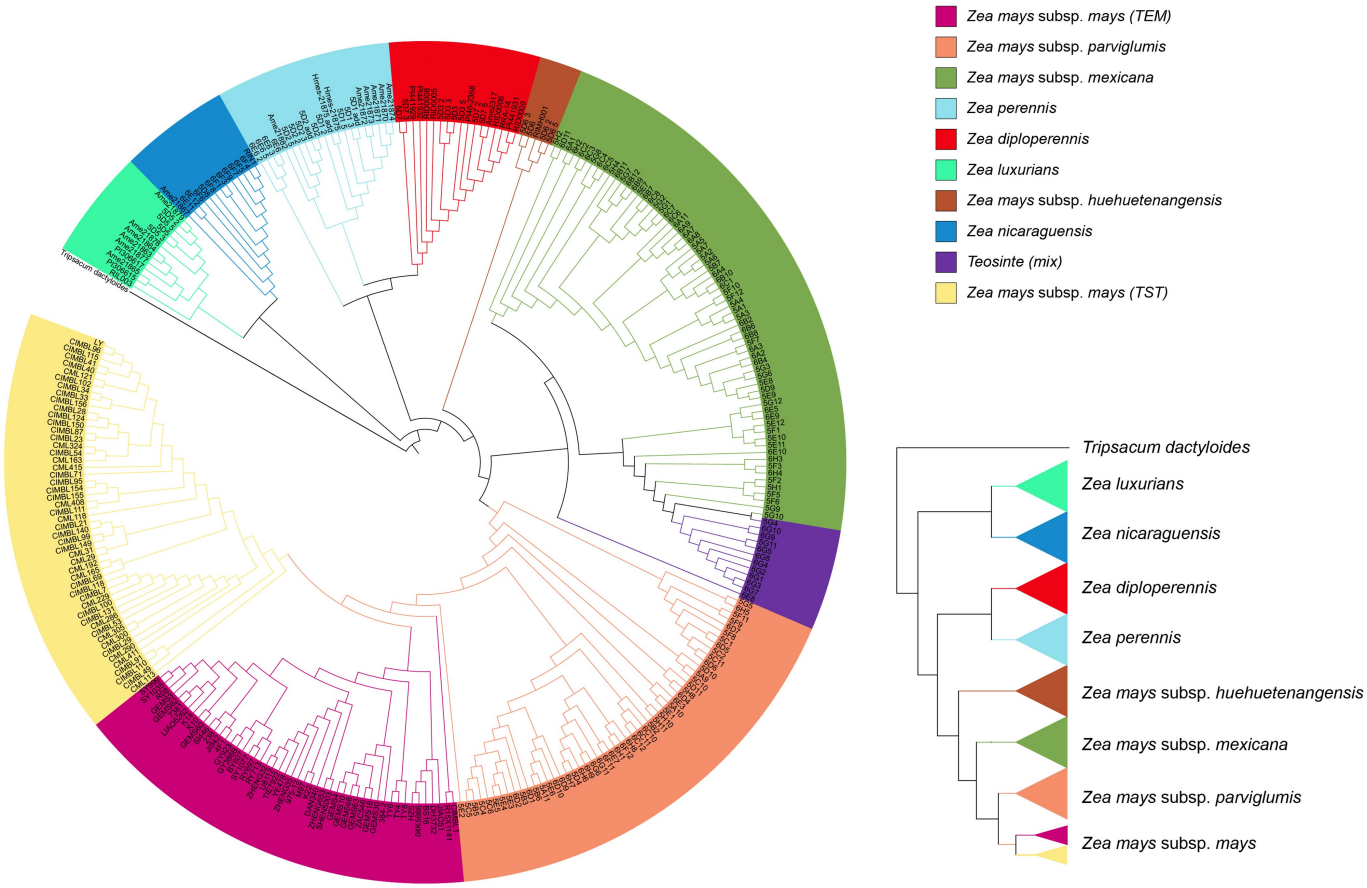
The phylogenetic tree constructed using MIKE for 303 samples of the genus *Zea*

Additionally, 303 samples from the maize genus were also utilized reconstruct a phylogenetic tree for validating the robustness of MIKE. As showed in Fig. 4, using *Tripsacum dactyloides* as an outgroup, within this genus, *luxurians* and *nicaraguensis* diverged first, followed by *diploperennis* and *perennis*. Additionally, *huehuetenangensis* may represent a subspecies of *Zea mays*, with its divergence time and phylogenetic tree structure highly resembling those reported by Chen *et al.* (2022). It suggests that the results obtained using MIKE align with the accepted outcomes, reinforcing the validity of our findings.

Subsequently, we turned our attention to *Oryza sativa*, which is extensively cultivated and encompasses a greater variety of subspecies. We selected 67 rice accessions and the dataset included five subspecies and a relative, comprising five accessions of *O.sativa* tropical *japonica*, 22 of *O.sativa* temperate *japonica*, 13 of *O.rufipogon*, 1 of *O.sativa aromatic*, 19 of *O.sativa indica*, and 6 of *O.sativa aus*. As shown in Supplementary Fig. S17, the relationship between *O.sativa aus* and *O.rufipogon* is closer, and *O.sativa* tropical *japonica* diverged from *O.sativa* temperate *japonica*. *O.sativa indica* and *O.sativa* tropical *japonica* form independent branches. Meanwhile, the RF distance between the phylogenetic trees constructed by MIKE and CallsSNPs was determined to be 0.35 in Supplementary Fig. S8. Although the classification is correct, there is still room for improvement.

**Figure 5.**
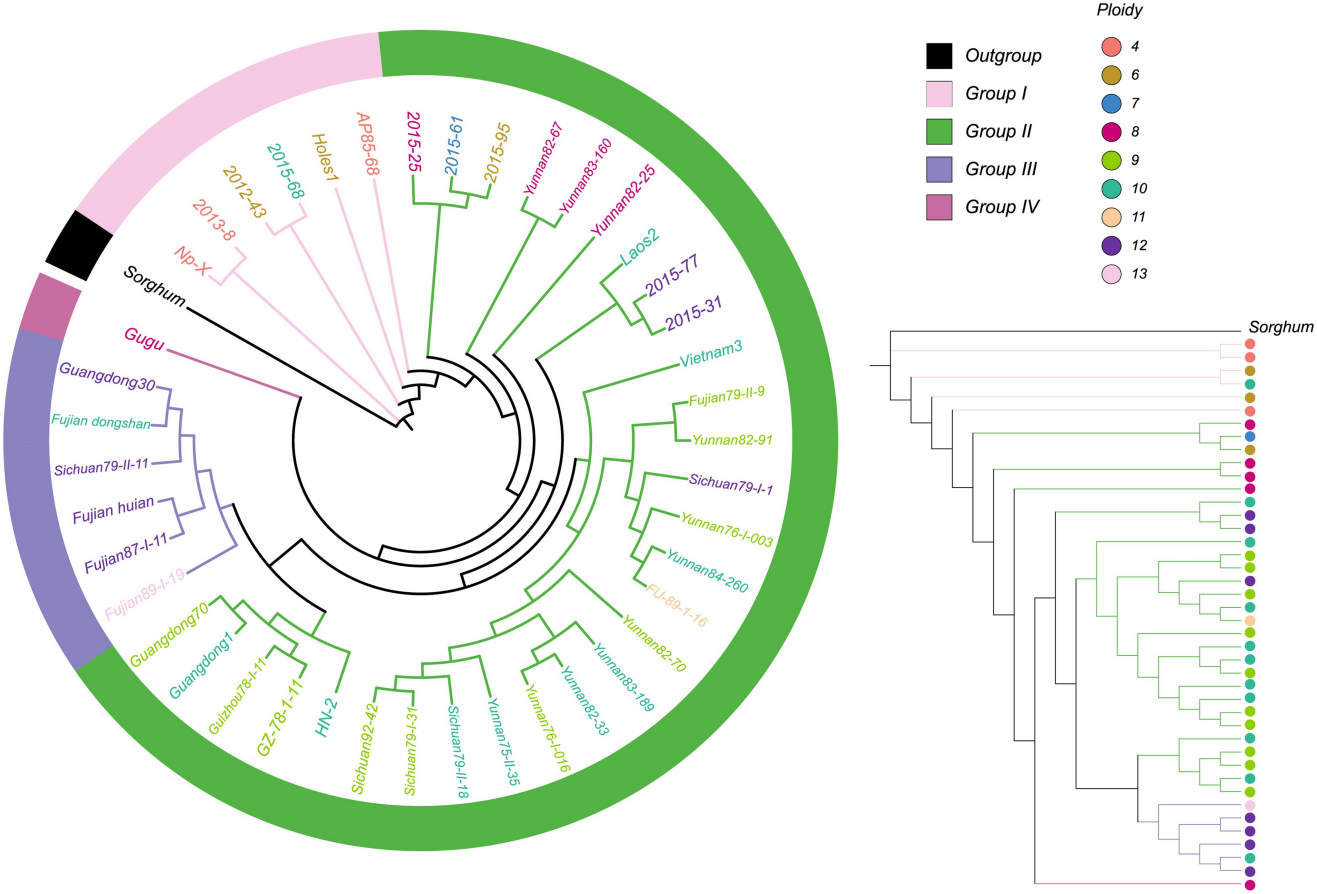
The phylogenetic tree constructed using MIKE for 42 samples of *S.spontaneum*

Our studies focused on species like *Ficus*, *Zea*, and *Oryza*, which are diploid. Subsequently, we shift attention to the issue of polyploid species, specifically examining sugarcane. Sugarcane is a polyploid species with varying ploidy levels, ranging from *n* = 4 to *n* = 16. We employed MIKE to construct phylogenetic trees for 42 individuals of *S.spontaneum* and its closely related species, *Sorghum*, serving as an outgroup in Fig. 5. The ploidies of these individuals range from *n* = 4 to *n* = 13. In the constructed phylogenetic tree, the topology reveals that Np-X is positioned closer to *Sorghum*. In connection with previous studies (Zhang *et al.* 2022), the 42 samples can be categorized into four groups. Tetraploid (*n* = 4) species are exclusively found in Group I. Hexaploid (*n* = 6) species are present in both Group I and Group II. Nonuploid (*n* = 9) species only occur in Group II, and decaploid (*n* = 10) is present in three of these groups. This categorization suggests that species within these four groups exhibit relatively distinct lineage differences.

## 4 Discussion

Traditional methods for the phylogenetic tree construction often demand substantial computational resources and typically require accurate genome assembly and annotation. Handling the genome directly from raw sequencing data not only circumvents these constraints but also reduces biases stemming from overreliance on specific reference genomes. Currently, available assembly- and alignment-free methods, such as Mash, Kssd, and BinDash, are not suitable for constructing phylogenetic trees. They are primarily designed for metagenomic datasets analysis (Ondov *et al.* 2016, Zhao 2019, Yi *et al.* 2021). While Mash mentions its application in phylogenetic tree construction, it emphasizes that it may not be suitable for data with significant differences in genome size. AAF is a tool explicitly designed for constructing phylogenetic trees using *k*-mers, but it is relatively slow (Fan *et al.* 2015). Skmer, which incorporates Mash for calculating the Jaccard coefficient and improves Mash’s evolutionary distance model, can be applied to construct phylogenetic trees, but it does not offer a significant improvement in speed (Sarmashghi *et al.* 2019). In contrast, MIKE demonstrates significant improvements in both speed and the accuracy of phylogenetic tree construction.

MIKE’s key improvement is its consideration of sequencing data specificity. It uses a grouping approach to preserve characteristic *k*-mers in sequencing data, greatly improving Jaccard coefficient estimation accuracy. Furthermore, it calculates a distance matrix using an evolutionary distance formula and constructs a phylogenetic tree using the BIONJ or NJ method. However, it is important to note that it constructs phylogenetic trees using a distance-based method, which can result in significant differences in tree topology compared to those constructed using maximum likelihood methods. And, based on the above results, it is evident that MIKE is more suitable for constructing phylogenetic trees at the genus level. Therefore, the choice between methods should be based on the specific research objectives. Despite the limitations imposed by *k*-mer and distance-based methods, MIKE’s efficiency and moderate memory usage still contribute significantly to the conservation of computational resources. It maintains an advantage in swiftly constructing phylogenetic trees, a feat that traditional methods cannot match.

We believe that the capabilities of MIKE extend beyond phylogenetic tree construction and can be applied in various other domains. Comparing unknown samples with known samples, and calculating the similarity between unknown and known samples, can effectively classify unknown biological samples into specific categories. Sample clustering, which groups similar samples and data points together, provides better insights into the underlying structure and patterns within the data. The Jaccard coefficient can be employed in clustering algorithms as a metric for identifying similarity. These represent potential applications and prospects. The accuracy and efficiency of MIKE make it a versatile tool for a wide range of applications.

## Supplementary Material

btae154_Supplementary_Data

## Data Availability

MIKE is publicly available at https://github.com/Argonum-Clever2/mike.git under the GPL-3.0 license. KMC is available at https://github.com/refresh-bio/KMC.git. Fasttree is available at the http://www.microbesonline.org/fasttree. Art_illumina is available at https://www.niehs.nih.gov/research/resources/software/biostatistics/art/. Seqkit is available at https://github.com/shenwei356/seqkit/releases/tag/v2.5.1. Mash is available at https://github.com/marbl/Mash.git. BinDash is available at https://github.com/zhaoxiaofei/BinDash.git. Kssd is avalibale at https://github.com/yhg926/public_kssd.git. We used gatk4 (Poplin *et al.* 2017) to callSNPs, and gatk4 is available at https://github.com/broadinstitute/gatk/releases. Bwa (Li 2013) is available at https://github.com/lh3/bwa. Samtools (Danecek *et al.* 2021) is available at https://github.com/samtools/samtools. Rstudio was employed to construct phylogenetic trees using R version 4.2.2. Several R packages were utilized in this process, including ape (Paradis and Schliep 2019), phytools (Revell 2012), and TreeDist. The ape and phytools packages were used to build the phylogenetic tree, while the TreeDist package was used to calculate the RF (Robinson–Foulds) distance. The visualization and presentation of the phylogenetic tree was accomplished using iTOL (Interactive Tree of Life) (Letunic and Bork 2021). The *Drosophila melanogaster* genome assembly is obtained from InsectBase-Download (insect-genome.com) (Yin *et al.* 2016). The 26 species genome assemblies (Cannarozzi *et al.* 2014, Ray *et al.* 2021, Wötzel *et al.* 2022) are obtained from NCBI database. The 141 *Ficus* sequencing reads are obtained from the GSA database in BIG Data Center (https://bigd.big.ac.cn/gsa/) under project number GSA: PRJCA002187. The 303 *Zea* sequencing reads are obtained from NCBI Sequence Read Archive with the accession code PRJNA641489, PRJNA816255, and PRJNA531553. The 67 *Orzya* genome assemblies are available at the RicePanGenome database (http://www.ncgr.ac.cn/RicePanGenome) (Zhao *et al.* 2018). The 42 *Saccharum spontaneum* sequencing data are obtained from CNCB under project number PRJNA721787. All the information for the samples can be found in Supplementary File.
