# Supplementary material for: MIKE: an ultrafast, assembly-, and alignment-free approach for phylogenetic tree construction
Source: Bioinformatics. 2024 Mar 28;40(4):btae154. doi: 10.1093/bioinformatics/btae154 (PMC10990684; doi:10.1093/bioinformatics/btae154)
Supplement: btae154_Supplementary_Data [file btae154_supplementary_data.zip › Supplementary Material.pdf]

# Supplementary Material

# MIKE: an ultrafast, assembly and alignment-free approach for phylogenetic tree construction

Fang Wang[1,2], Yibin Wang[2], Xiaofei Zeng[3], Shengcheng Zhang[2], Jiaxin Yu[2], Dongxi Li[1,*], Xingtan Zhang[2,*]

[1]College of Computer Science and Technology, Taiyuan University of Technology, Taiyuan, Shanxi 030024, China

[2]National Key Laboratory for Tropical Crop Breeding, Shenzhen Branch, Guangdong Laboratory for Lingnan Modern Agriculture, Genome Analysis Laboratory of the Ministry of Agriculture, Agricultural Genomics Institute at Shenzhen, Chinese Academy of Agricultural Sciences, Shenzhen, Guangdong 518120, China

[3]Department of Human Cell Biology and Genetics, Joint Laboratory of Guangdong-Hong Kong Universities for Vascular Homeostasis and Diseases, School of Medicine, Southern University of Science and Technology, Shenzhen, Guangdong 508055, China

*Correspondence, Email: zhangxingtan@caas.cn

*Correspondence, Email: dxli0426@126.com

**Appendix A: Supplementary method details and commands**

Here we provide the exact procedures and commands that we used to run external methods throughout our experiments.

Simulated sequencing data using ART

To simulate short reads with length $\ell = 150$ with default error profiles of Illumina HiSeq2500.

art _ illumina − ss HS25 − i FASTA _ FILE − p − l 150 − f SEQUENCE_COVERAGE − m 200 − s 10
− o FASTQ_FILE

To sample from the 100 x sequencing data at varied sequence coverage

seqkit − p PROPOTION − s INPUT _ FILE − o OUTPUT _ FILE

Processing Input Files with KMC

kmc − k21 − t10 INPUT _ FILE OUTPUT_FILE

Computing the Jaccard coefficient using different methods.

bindash sketch − nthreads = 10 − outfname = OUTPUT _ FILE INPUT_FILE
bindash dist OUTPUT _ FILE OUTPUT_FILE

mash sketch − p 10 − k 21 − s 1048576 INPUT_FILE OUTPUT_FILE
mash dist OUTPU _ FILE OUTPUT _ FILE

kssd dist − p 10 PATH/L3K10. shuf − r INPUT _ DIR − o OUTPUT_DIR
kssd dist − R OUTPUT _ FILE − o OUTPUT _ FILE OUTPUT _ DIR

CallSNPs and Computing the distance matrix

trimmomatic PE − threads 20 − phred33 INPUT_FILE OUTPUT_FILE ILLUMINACLIP: PATH/ TruSeq3 − PE
− 2. fa: 2: 30: 10: 8: True SLIDINGWINDOW: 5: 15 LEADING: 5 TRAILING: 5 MINLEN: 50
bwa mem − t 20 − M − Y
− R ′@RD\tID: ID\tSM: SM\tPL: ILLUMINA
\tLB: WES′ REFERENCE_GENOME INPUT_FILE | samtools view − Sb − > OUTPUT_FILE
samtools sort − @ 20 − o OUTPUT_FILE − i INPUT_FILE
gatk MarkDuplicates − I INPUT_FILE − o OUTPUT_FILE − M OUTPUT_FILE
samtools index INPUT_FILE
gatk CreateSequenceDictionary − R REFERENCE_FILE − o OUTPUT_FILE
gatk HaplotypeCaller − R REFERENCE_FILE − −emit − ref − confidence GVCF − I INPUT_FILE
− o OUTPUT_FILE
gatk GenotypeGVCFs − R REFERENCE_FILE − V INPUT_FILE − o OUTPUT_FILE
gatk CombineGVCFs − R REFERENCE_FILE − V INPUT_FILE − o OUTPUT_FILE

## Appendix B: Supplementary figures

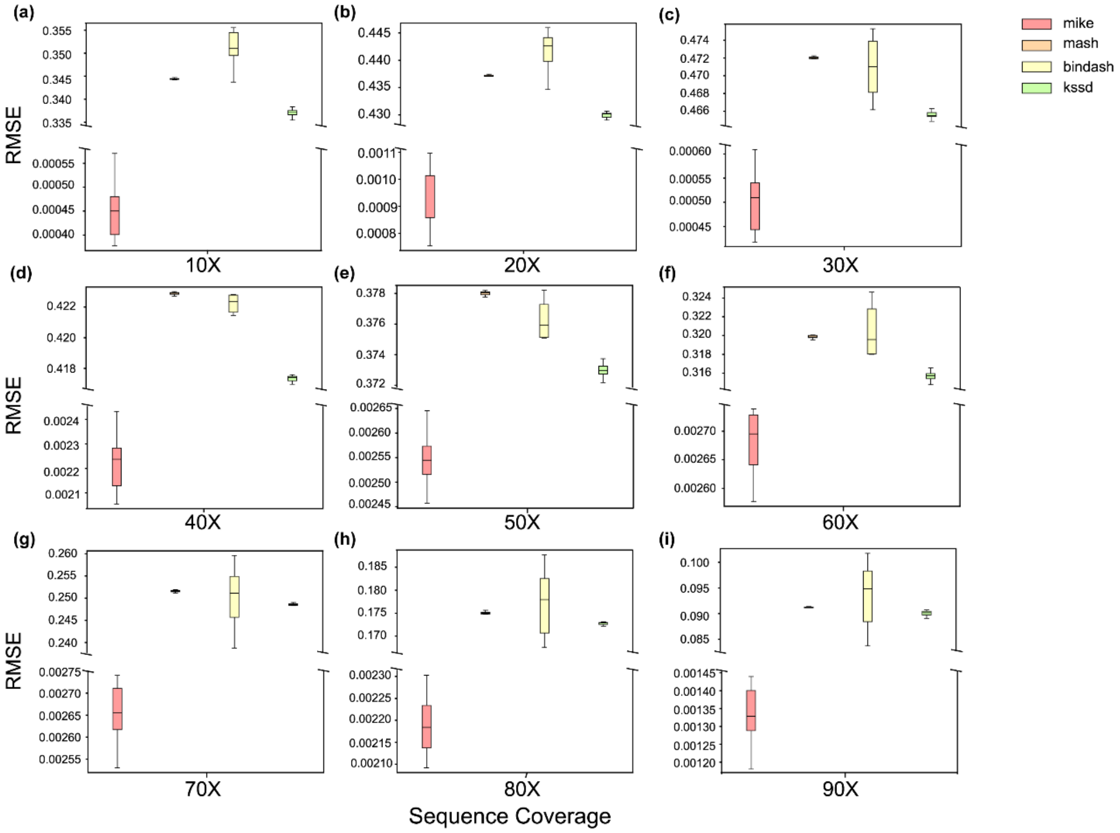


**Fig. S1. The box plots of the Root Mean Square Error (RMSE) obtained using different method at Equivalent Sequence coverage.** (a)-(i) The RMSE of 10 sets of replicates pairwise at 10 x ~ 90 x sequencing coverage.

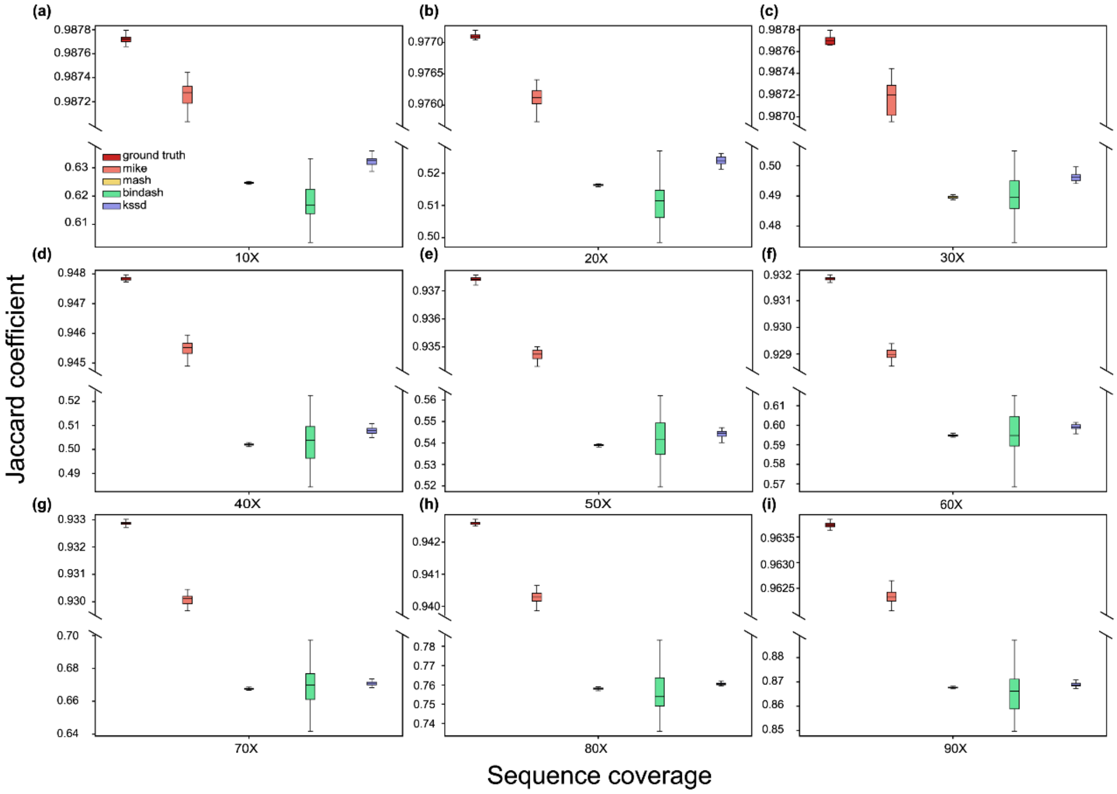


**Fig. S2. The box plots of the Jaccard coefficient between the ground truth and four approaches estimated values at same sequence coverage.** (a)-(i) The Jaccard coefficient of 10 sets of replicates pairwise at 10 x ~ 90 x sequencing coverage.

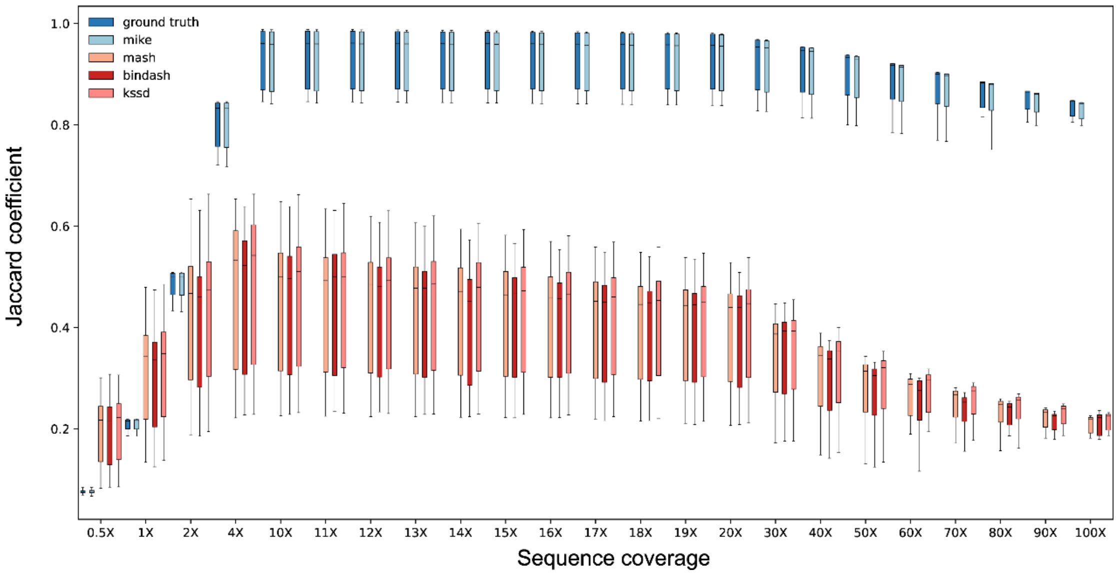


**Fig. S3. The box plots of the Jaccard coefficient between the ground truth and four approaches estimated values at various sequence coverage.**

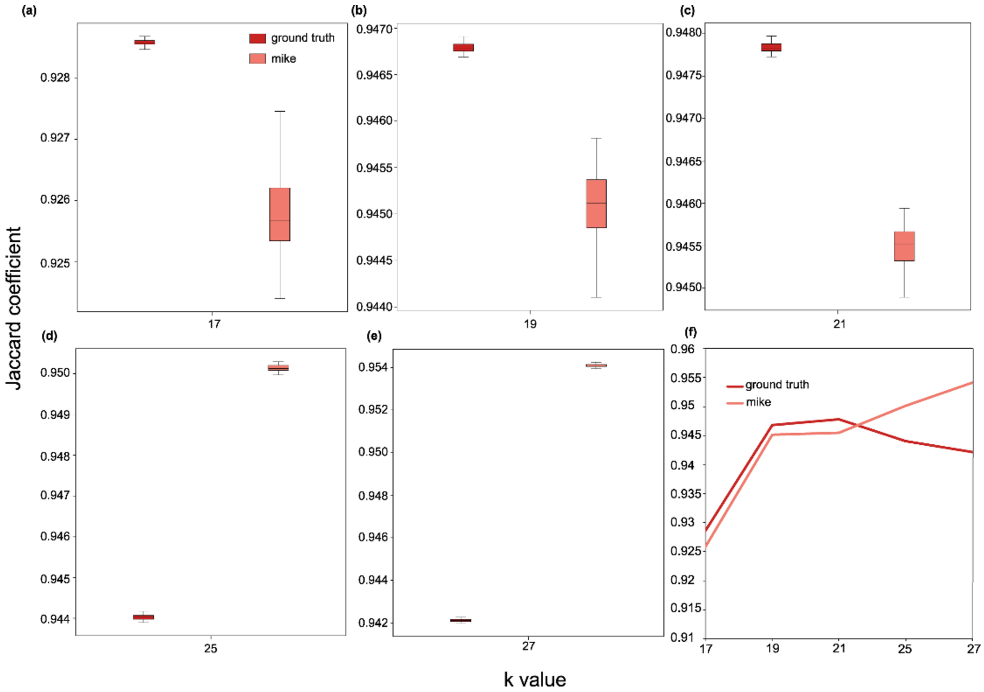


**Fig. S4. The box plots of the Jaccard coefficient between the ground truth and MIKE estimated values at various k values.**

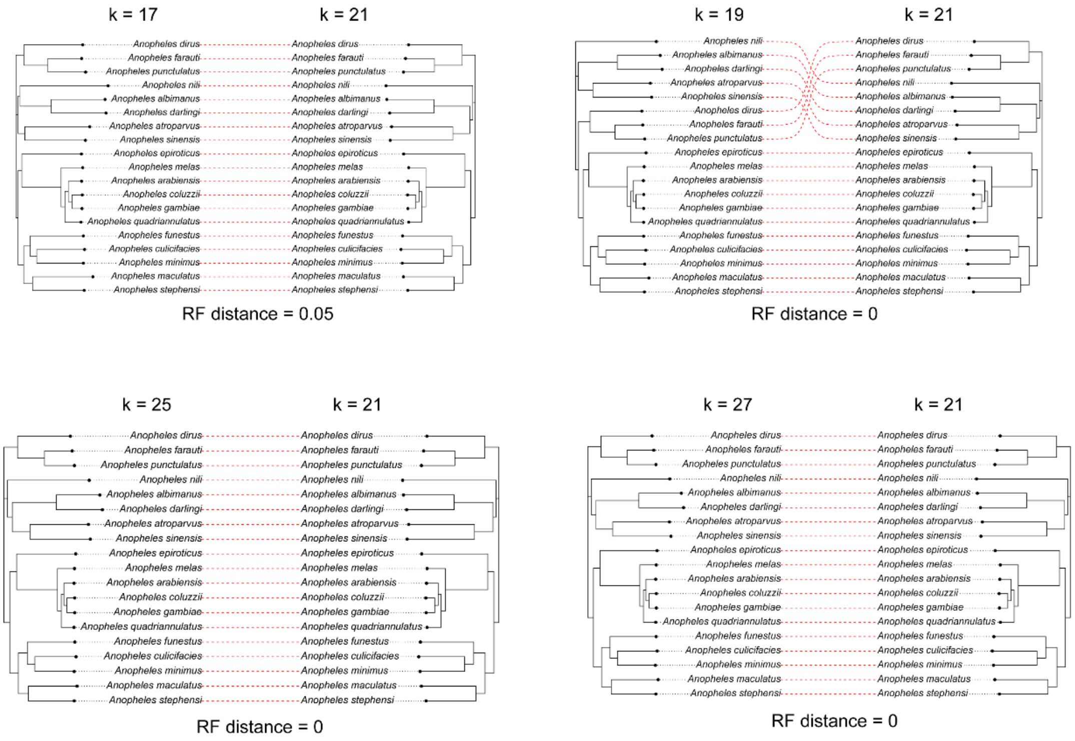


**Fig. S5. The Impact of Different k Values on Constructing Phylogenetic Trees.**

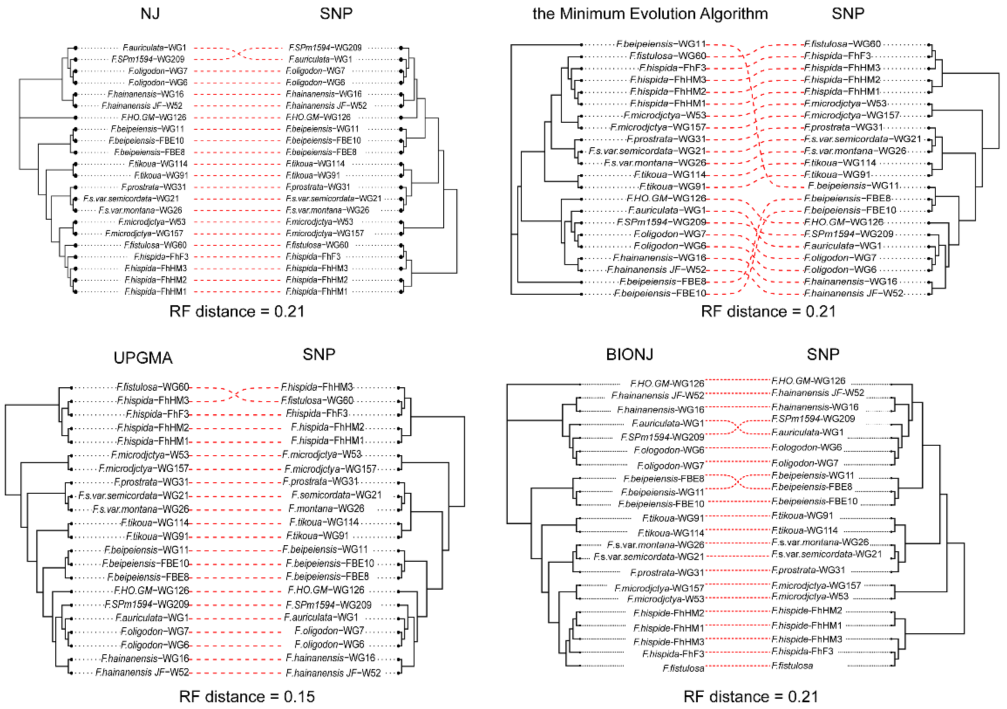


**Fig. S6. The phylogenetic trees construction of samples from 22 *Ficus* genera using different methods.**

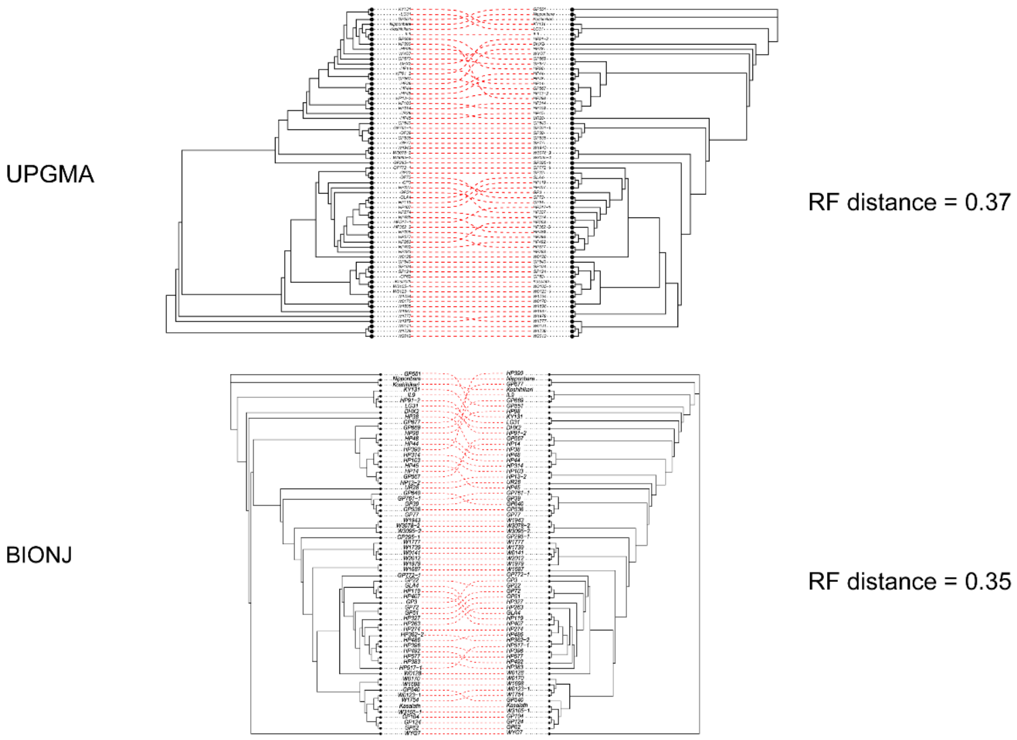


**Fig. S7. The phylogenetic trees construction of samples from 67 rice using UPGMA and BIONJ, respectively.**

NJ

RF distance = 0.35

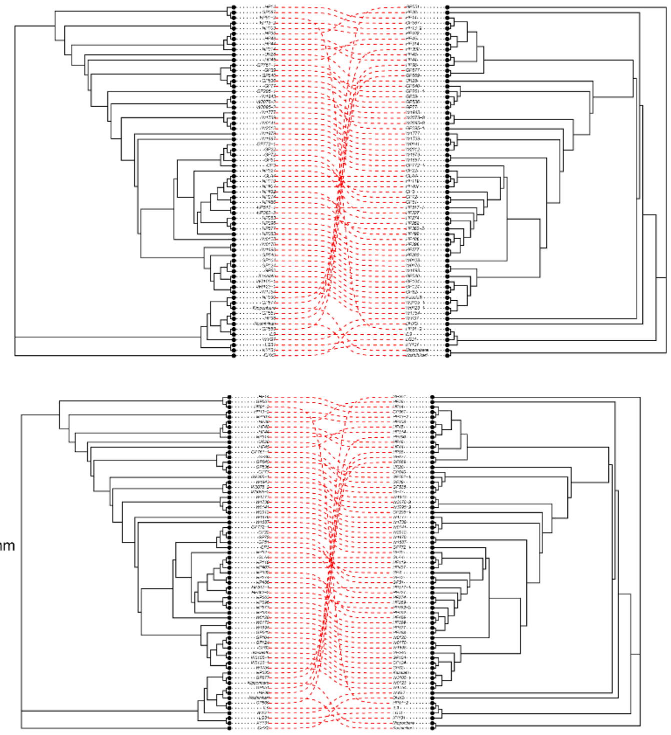

the Minimum Evolution Algorithm

RF distance = 0.37

**Fig. S8. The phylogenetic trees construction of samples from 67 *Oryza* genus using NJ and the Minimum Evolution Algorithm, respectively.**

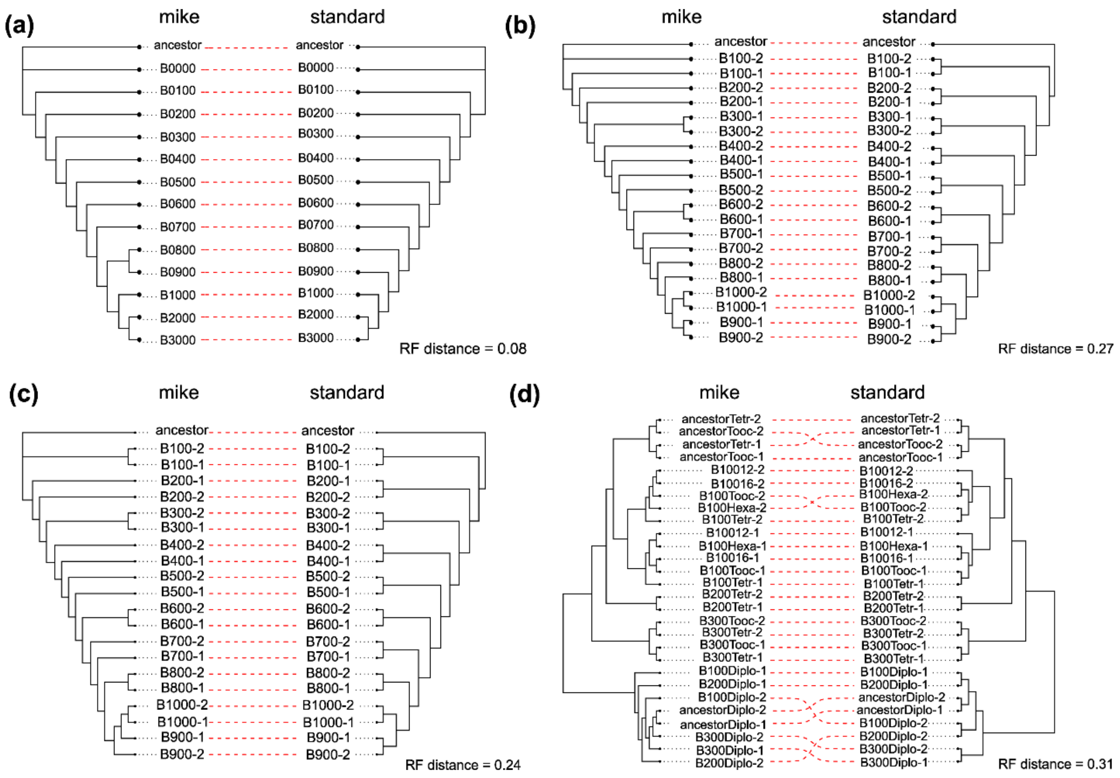


**Fig. S9. The Phylogenetic Trees Constructed from Simulated Data using MIKE.** (a) The phylogenetic tree constructed for haploid simulated data and compared with the standard tree. (b) The phylogenetic tree constructed for autotetraploid simulated data and compared with the standard tree. (c) The phylogenetic tree constructed for allopolyploid simulated data and compared with the standard tree. (d) The phylogenetic tree constructed for polyploid simulated data and compared with the standard tree. In this simulation, ancestors with x = 2 and x = 4 were generated. The x = 4 underwent WGD in a certain generation, resulting in x = 8, which then underwent further WGD to become x = 16. The x = 4 and x = 8 underwent hybridization, producing x = 6, which subsequently doubled to become x = 12.

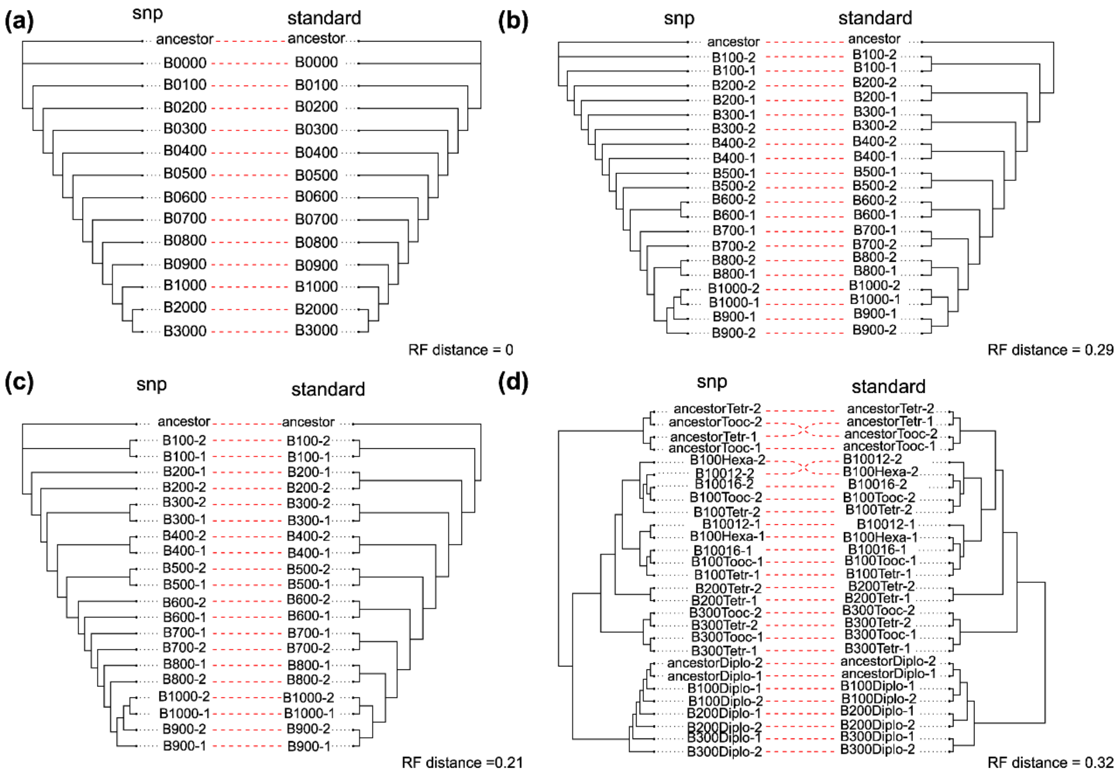


**Fig. S10. The Phylogenetic Trees Constructed from Simulated Data using CallSNPs.** (a) the phylogenetic tree constructed for haploid simulated data and compared with the standard tree. (b) the phylogenetic tree constructed for autotetraploid simulated data and compared with the standard tree. (c) the phylogenetic trees constructed for allopolyploid simulated data and compared with the standard tree. (d) the phylogenetic trees constructed for polyploid simulated data and compared with the standard tree. In this simulation, ancestors with x = 2 and x = 4 were generated. The x = 4 underwent WGD in a certain generation, resulting in x = 8, which then underwent further WGD to become x = 16. The x = 4 and x = 8 underwent hybridization, producing x = 6, which subsequently doubled to become x = 12.

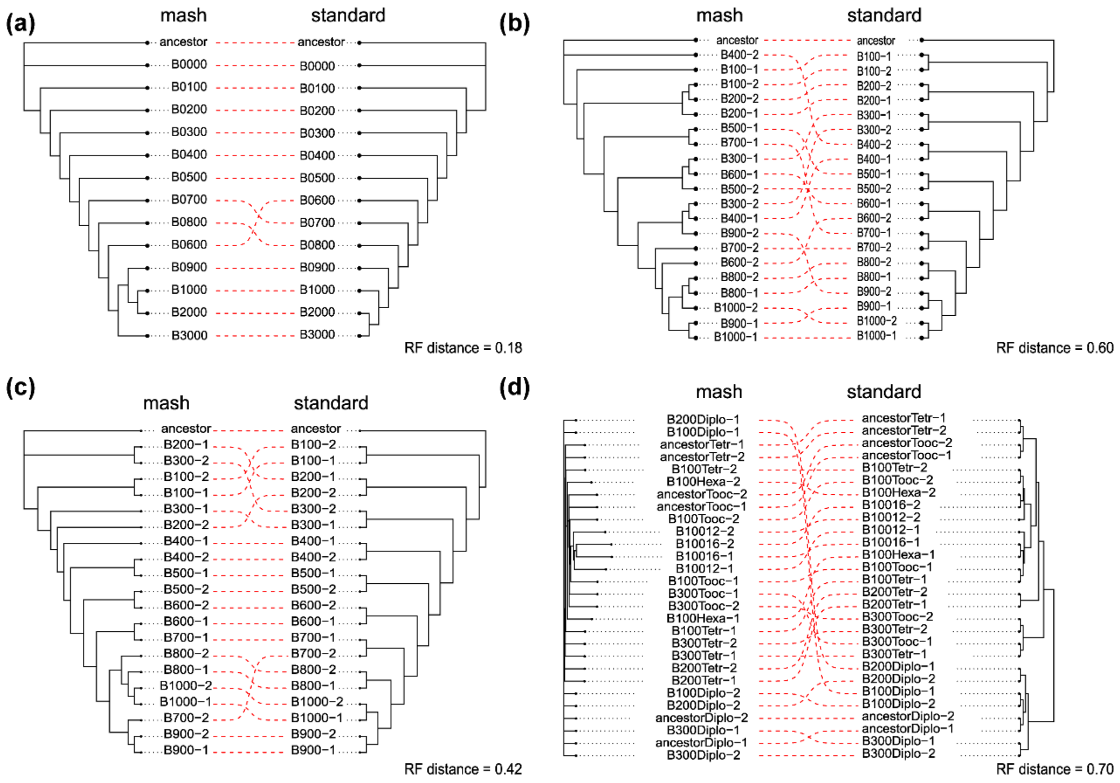


**Fig. S11. The Phylogenetic Trees Constructed from Simulated Data using Mash.** (a) The phylogenetic tree constructed for haploid simulated data and compared with the standard tree. (b) The phylogenetic tree constructed for autotetraploid simulated data and compared with the standard tree. (c) The phylogenetic trees constructed for allopolyploid simulated data and compared with the standard tree. (d) The phylogenetic trees constructed for polyploid simulated data and compared with the standard tree. In this simulation, ancestors with x = 2 and x = 4 were generated. The x = 4 underwent WGD in a certain generation, resulting in x = 8, which then underwent further WGD to become x = 16. The x = 4 and x = 8 underwent hybridization, producing x = 6, which subsequently doubled to become x = 12.

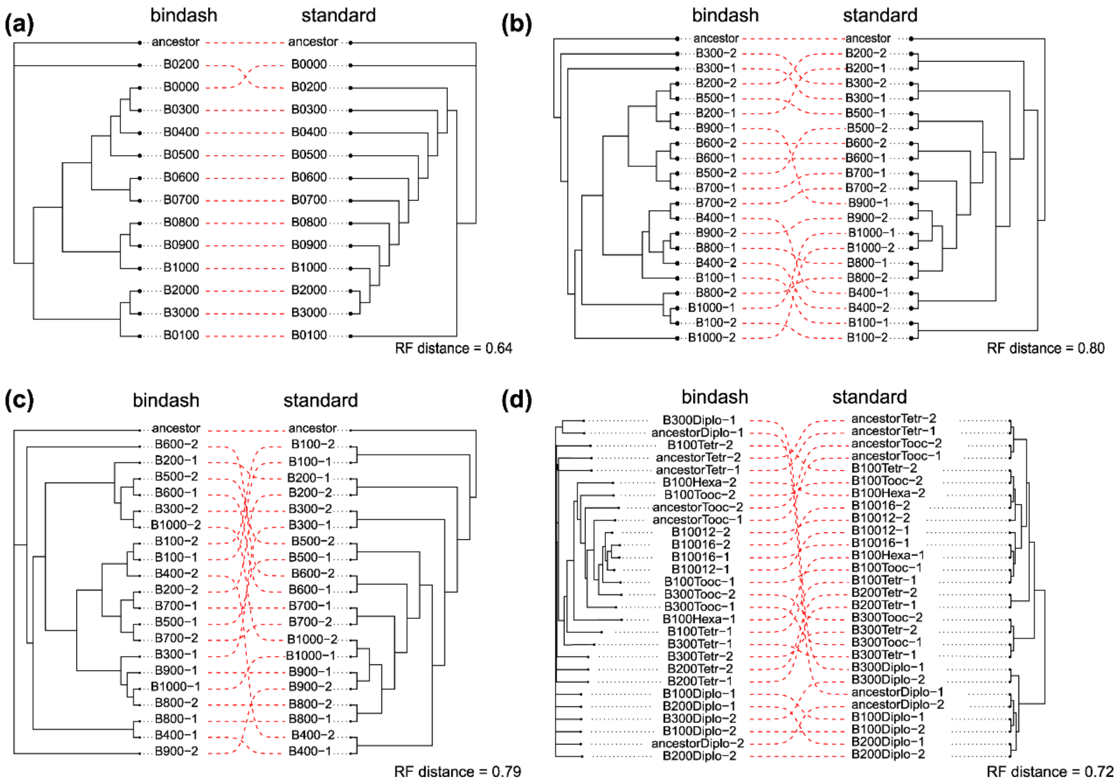


**Fig. S12. The Phylogenetic Trees Constructed from Simulated Data using BinDash.** (a) The phylogenetic tree constructed for haploid simulated data and compared with the standard tree. (b) The phylogenetic tree constructed for autotetraploid simulated data and compared with the standard tree. (c) The phylogenetic trees constructed for allopolyploid simulated data and compared with the standard tree. (d) The phylogenetic trees constructed for polyploid simulated data and compared with the standard tree. In this simulation, ancestors with x = 2 and x = 4 were generated. The x = 4 underwent WGD in a certain generation, resulting in x = 8, which then underwent further WGD to become x = 16. The x = 4 and x = 8 underwent hybridization, producing x = 6, which subsequently doubled to become x = 12.

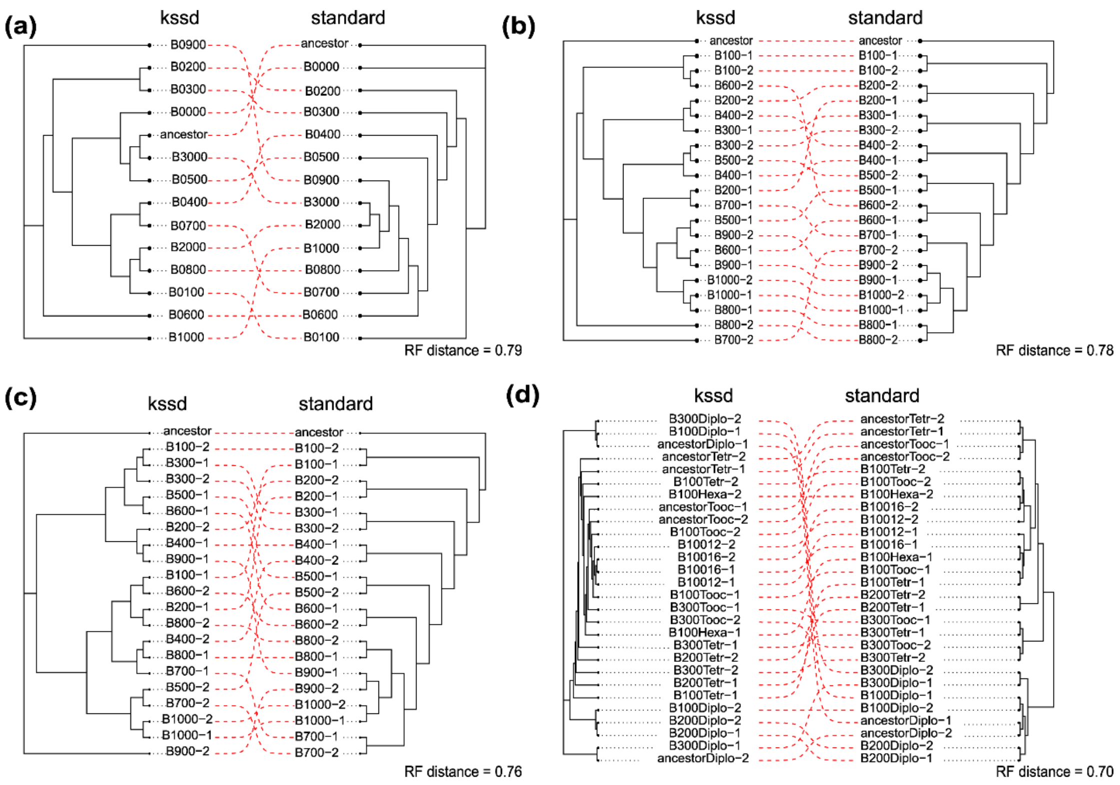


**Fig. S13. The Phylogenetic Trees Constructed from Simulated Data using Kssd.** (a) The phylogenetic tree constructed for haploid simulated data and compared with the standard tree. (b) The phylogenetic tree constructed for autotetraploid simulated data and compared with the standard tree. (c) The phylogenetic trees constructed for allopolyploid simulated data and compared with the standard tree. (d) The phylogenetic trees constructed for polyploid simulated data and compared with the standard tree. In this simulation, ancestors with x = 2 and x = 4 were generated. The x = 4 underwent WGD in a certain generation, resulting in x = 8, which then underwent further WGD to become x = 16. The x = 4 and x = 8 underwent hybridization, producing x = 6, which subsequently doubled to become x = 12.

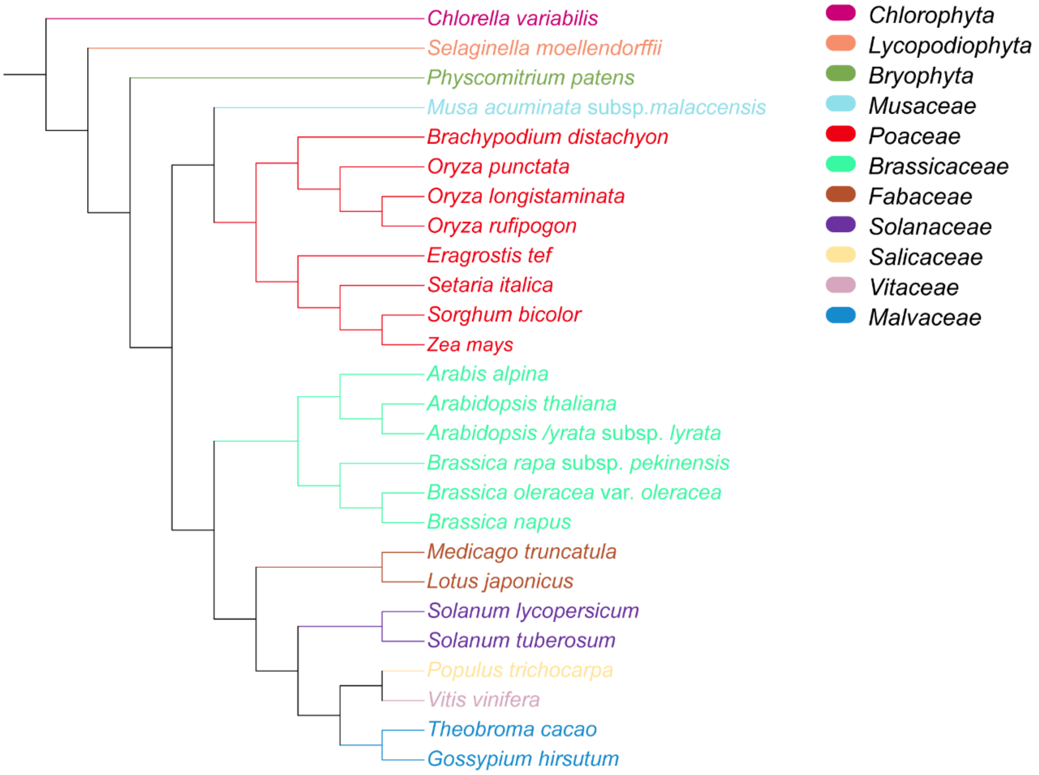


**Fig. S14. The phylogenetic tree constructed by MIKE**

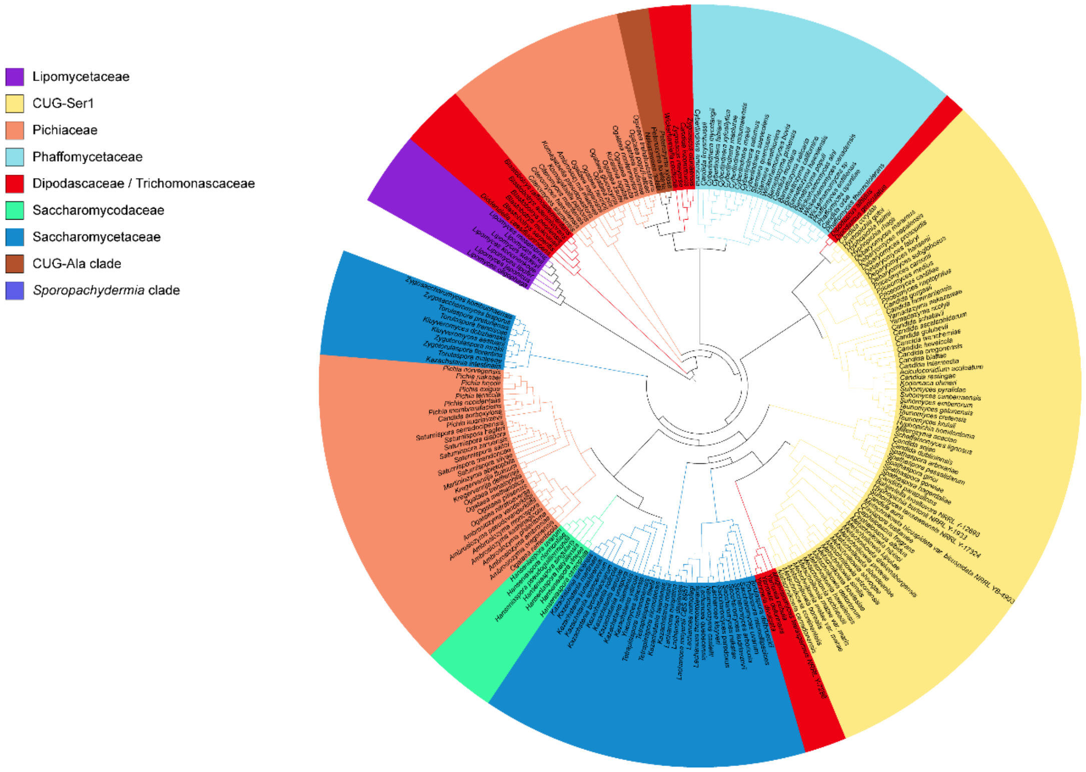


**Fig. S15. The phylogenetic tree of 238 yeast species.**

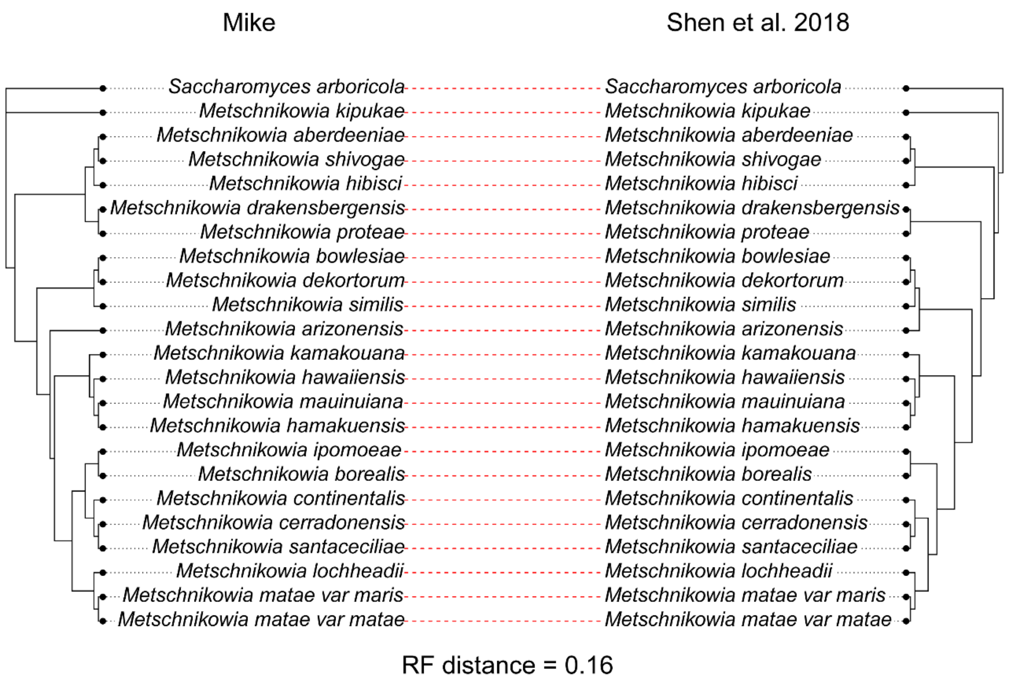


**Fig. S16. The phylogenetic tree constructed by MIKE was compared to the work of David Dylu et al. with a focus on the Metschnikowia family.**

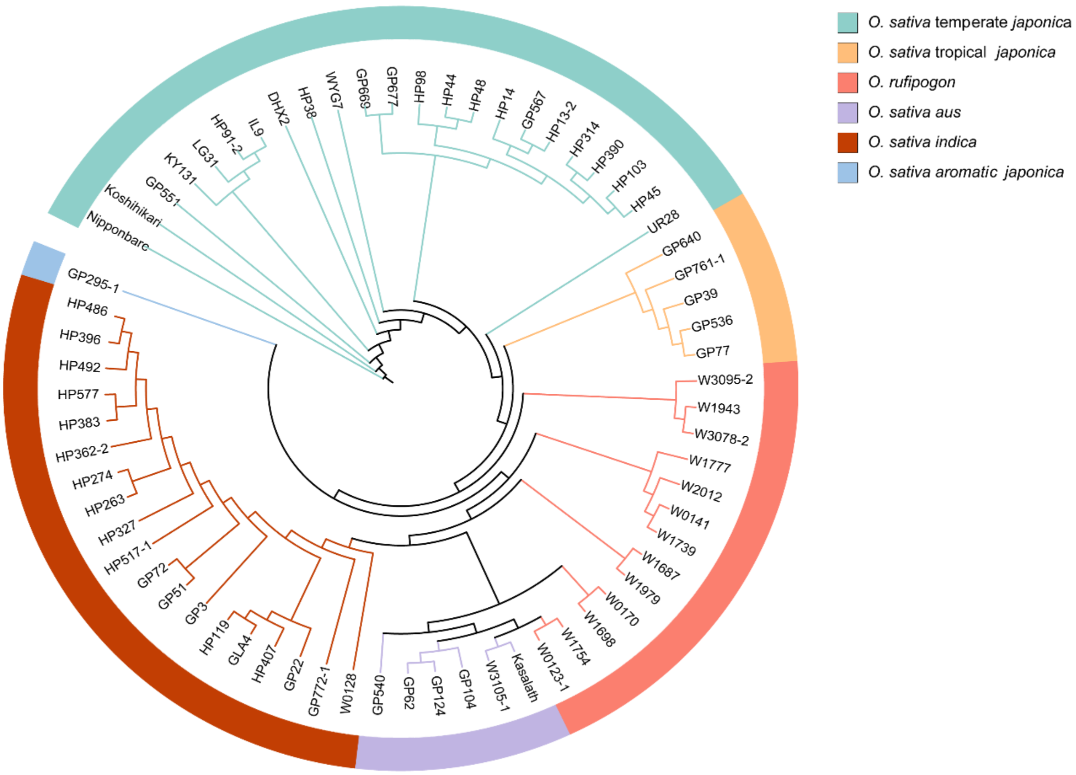


**Fig. S17. The phylogenetic tree of the genus Oryza.**
